# Chloroplasts of Salt-Grown *Arabidopsis* Seedlings Are Impaired in Structure, Genome Copy Number and Transcript Levels

**DOI:** 10.1371/journal.pone.0082548

**Published:** 2013-12-05

**Authors:** Petra Peharec Štefanić, Tal Koffler, Guy Adler, Dudy Bar-Zvi

**Affiliations:** 1 Department of Life Sciences and Doris and Bertie Black Center for Bioenergetics in Life Sciences, Ben-Gurion University of the Negev, Beer-Sheva, Israel; 2 Department of Molecular Biology, Division of Biology, Faculty of Science, University of Zagreb, Horvatovac 102a, Zagreb, Croatia; University of California - Davis, United States of America

## Abstract

The chloroplast is the most prominent and metabolically active plastid in photosynthetic plants. Chloroplasts differentiate from proplastids in the plant meristem. Plant plastids contain multiple copies of a small circular genome. The numbers of chloroplasts per mesophyll cell and of plastid genome copies are affected by developmental stage and environmental signals. We compared chloroplast structure, gene expression and genome copy number in *Arabidopsis* seedlings germinated and grown under optimal conditions to those in seedlings germinated and grown in the presence of NaCl. Chloroplasts of the NaCl-grown seedlings were impaired, with less developed thylakoid and granum membranes than control seedlings. In addition, chloroplasts of salt-grown *Arabidopsis* seedlings accumulated more starch grains than those in the respective control plants. Steady-state transcript levels of chloroplast-encoded genes and of nuclear genes encoding chloroplast proteins were reduced in salt-grown seedlings. This reduction did not result from a global decrease in gene expression, since the expression of other nuclear genes was induced or not affected. Average cellular chloroplast genome copy number was reduced in salt-grown seedlings, suggesting that the reduction in steady-state transcript levels of chloroplast-encoded genes might result from a decrease in template DNA.

## Introduction

Salt stress is a major abiotic stress that limits plant growth and productivity worldwide [1]. Exposure of plants to high salt concentrations results in primary salt stress, composed of osmotic stress and ion toxicity, and secondary oxidative stress [2]. Plants exposed to salt stress respond with global changes in cellular activity, including physiological and molecular changes, one of the main effects being stomatal closure. Photosynthesis is one of the primary cellular activities affected by salt stress [3]. The chloroplast is one of the primary organelles affected by salt stress. This results in a decrease in carbon-fixation rates, concomitant with reactive oxygen species production. Although the chloroplast contains its own genome, its coding capacity is rather limited (ca. 100–250 genes). Thus, most chloroplast proteins are encoded by the nucleus, and are post-translationally imported into the chloroplast. As a result, most studies on the impact of salt stress on gene expression are carried out on nucleus-encoded genes (reviewed by [4]). 

Chloroplasts are highly structured plastids with a characteristic extensive thylakoid-membrane network. Shoot apical meristems are believed to contain proplastids, lacking thylakoids and chlorophyll-binding proteins, which differentiate into chloroplasts very early in the development of leaf primordia [5–8]. It has been recently shown that shoot apex meristem cells contain proplastids and chloroplasts at various developmental stages [9]. The plastid genome is a circular 100–200 kb DNA molecule arranged in two regions of unique sequences separated by two inverted repeats harboring, in addition, ribosomal DNA genes [10]. Chloroplasts contain multiple copies of genomic DNA. For example, it is estimated that the diploid *Arabidopsis* cell contains approximately 560 copies of the plastid genome [11]. Genome copy number increases with leaf development, and is reduced in mature chloroplasts or senescing leaves [12,13]. Chloroplast size and number are also affected by environmental factors, such as light intensity and the availability of water and minerals [14,15]. Unlike chloroplasts in true leaves that develop from meristematic proplastids, cotyledon chloroplasts develop from etioplasts that are present in the embryo, and are rapidly converted to chloroplasts upon exposure to light [8]. Cotyledon chloroplasts resemble the true chloroplasts of young leaves containing a less extensive thylakoid-membrane system than that of mature leaf chloroplasts [16]. 

In this study, we characterized the effects of salinity on chloroplast morphology and on gene expression of chloroplast-encoded genes in 2-week-old *Arabidopsis* seedlings that were germinated and grown in the presence of 100 mM NaCl. Seedlings developed in NaCl-containing medium developed thicker leaves with smaller surface area. Chloroplasts developed in the presence of salt were swollen, with less developed granum structures and more starch accumulation than chloroplasts in *Arabidopsis* seedlings grown without salt. Steady-state transcript levels of plastid-encoded genes, as well as of nuclear genes encoding chloroplast proteins, were lower in salt-grown seedlings than in seedlings grown under non-stressed conditions. Quantification of the chloroplast genome showed that the number of plastid DNA copies per haploid nuclear genome is reduced in salt-grown seedlings, suggesting that the reduction in transcripts of chloroplast-encoded genes may result from a reduction in template quantity. 

## Materials and Methods

### Plant material and growth conditions

*Arabidopsis thaliana* (Col) seedlings were surface-sterilized and cold-treated as previously described [17]. Seeds were plated in Petri dishes containing 0.5% agar-solidified 0.5 strength MS salt mixture (Duchefa), and grown under 12/12 h light/dark circadian regime as described previously [17]. Where indicated, growth medium also included 100 mM NaCl. Unless otherwise specified, 2-week-old seedlings were used for all studies. 

### Chlorophyll assay

Leaf tissue was extracted overnight with 90% (v/v) acetone, absorbance was measured at different wavelengths (661 and 644 nm) after the tissue had been bleached, and the concentrations of chlorophyll a and b were calculated [18].

### Light and transmission electron microscopy

Tissue was fixed with 1% (w/v) glutaraldehyde in 50 mM cacodylate buffer (pH 7.2) for 30 min at 4 °C, and washed for 10 min with ice cold 50 mM cacodylate buffer. Tissue was then post-fixed with 1% (w/v) osmium tetroxide in the same buffer for 1 h at 4 °C, followed by 10 min wash in ice-cold water. After dehydration in a graded series of ethanol, the tissue was embedded in Araldite. Semi-thin sections (1 µm thick) of fixed material were stained with 2% (w/v) toluidine blue and examined under a light microscope. Ultrathin sections were stained with uranyl acetate and lead citrate and examined using a FEI Tecnai 12 G2 TWIN transmission electron microscope (Eindhoven, The Netherlands).

### Assays of RNA and DNA levels

Relative steady-state transcript levels were assayed by quantitative (q) RT-PCR as described previously [17,19–21]. RNA was isolated from seedlings using AurumTM Total RNA Mini Kit (Bio-Rad) according to the manufacturer's instructions. cDNA was synthesized from DNase-treated RNA with ABgene Reverse-iTTM 1st Strand Synthesis Kit using random decamer primers. Gene-specific primer sequences were designed by Primer-Express software Vers. 2.0 (Applied Biosystems). Where possible, one of the primers in each set was designed at an exon–exon border to reduce possible amplification from contaminating genomic DNA. All amplicon lengths were between 75 and 90 bp. Primer sequences are presented in Table S1 in File S1. Relative transcript levels were assayed by real-time qRT-PCR analysis using the 7300 Real-Time PCR System (Applied Biosystems), with 18S rRNA as the internal standard.

Genomic DNA was prepared from seedlings essentially as described previously [22]. Seedlings were homogenized in DNA extraction buffer (0.2 M Tris-HCl pH 7.5, 0.25 M NaCl, 25 mM EDTA, 0.5% (w/v) SDS). Homogenates were centrifuged for 5 min at 12,000 x g. The supernatant was removed to a new microcentrifuge tube to which an equal volume of isopropanol was added. Samples were incubated for 2 min at room temperature and centrifuged 5 min at 12,000 x g. Pellets were dried and resuspended in deionized water. DNA was quantified by qPCR using DNA templates. Primers designed for nuclear and plastid genes are listed in Table S2 in File S1. Relative plastid copy number was calculated by comparing ratios between plastid-encoded genes and the nuclear gene *WHIRLY1*. Other conditions were as described previously [17,19–21].

Each assay was performed in three biological replicates. 

## Results and Discussion

### Salt stress affects leaf morphology

**Figure 1:**
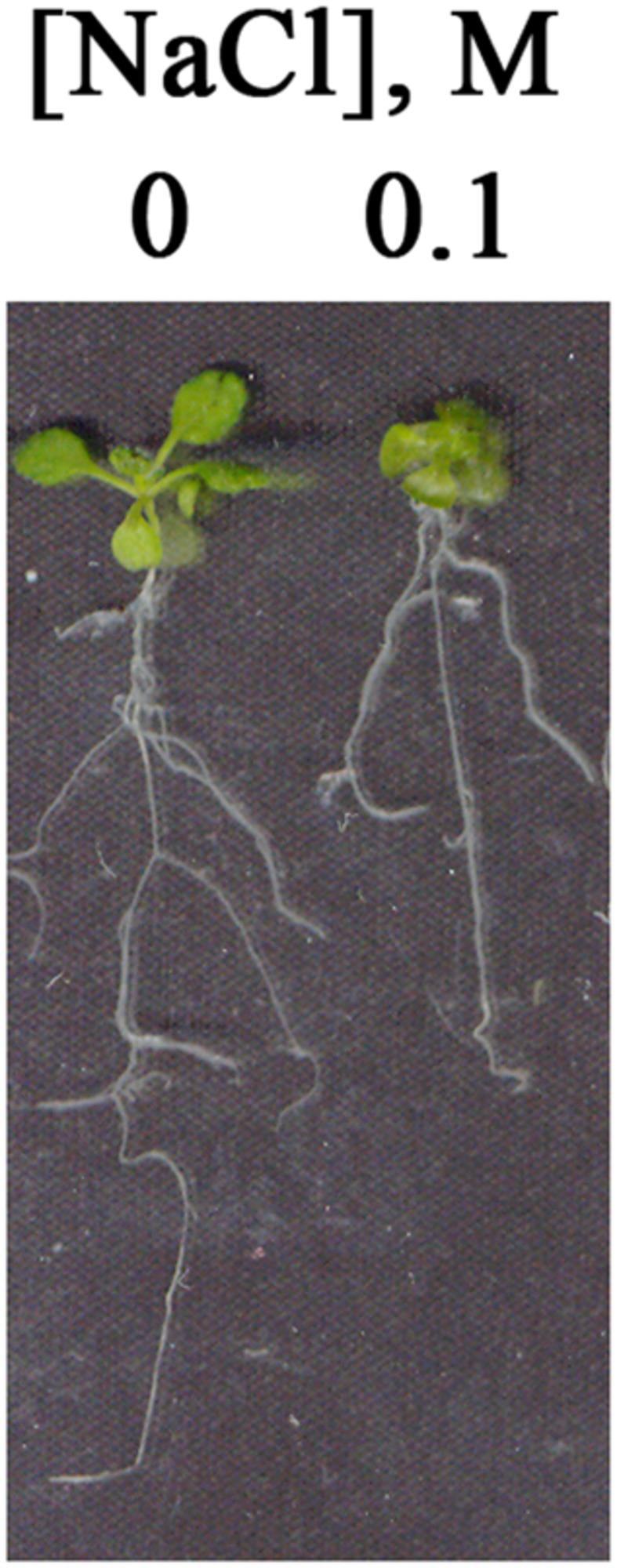
Two-week-old seedlings germinated and grown on agar-solidified medium with or without added NaCl. Surface-sterilized cold-treated seeds were sown on 0.5X MS, 0.5% sucrose and 0.5% agar containing 0 or 0.1 M NaCl.

**Figure 2:**
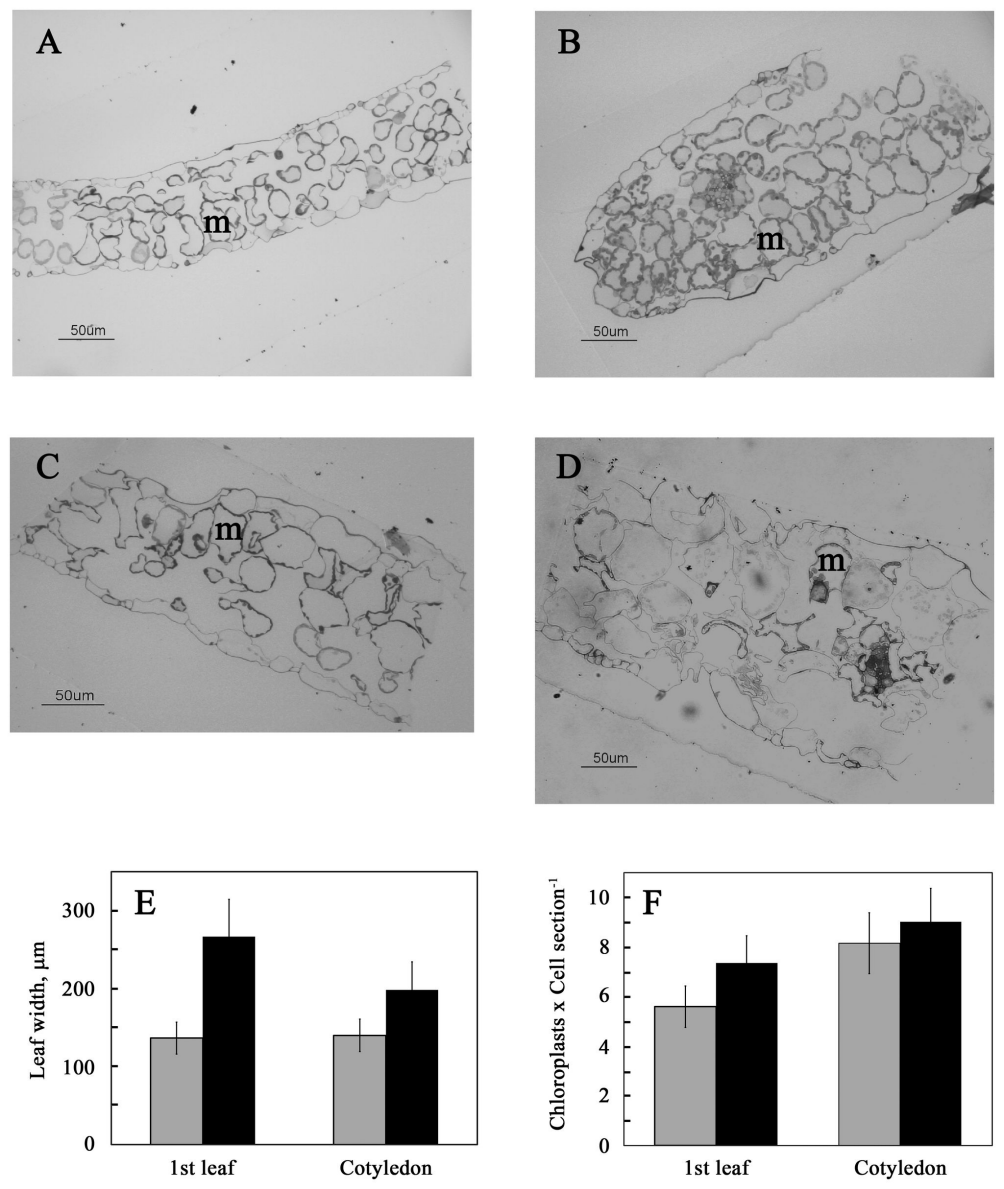
Sections of leaves and cotyledons of control and salt-treated seedlings. Two-week-old seedlings, germinated and grown as in Figure 1, were fixed and embedded as described in Materials and Methods. Semi-thin (1 µm) sections were stained with 2% toluidine blue and examined under a light microscope. (A) 1^st^ leaf of control plant. (B) 1^st^ leaf of salt-grown plant. (C) Cotyledon of control plant. (D) Cotyledon of salt-grown plant. m, mesophyll cells. (E and F) Leaf thickness and chloroplast count per cell, respectively, in semi-thin sections of 1^st^ leaves and cotyledons of control (gray bars) and salt-grown (black bars) seedlings. Data shown are average ± SE.

Seedlings germinated and grown in the presence of 0.1 M NaCl were smaller than those grown under non-stress conditions, with reduced size of all vegetative tissues (Figure 1). In addition, leaves and cotyledons of salt-grown seedlings were thicker than those of seedlings grown in the absence of salt (Figure 2A–E). Mesophyll cells were expanded in salt-grown plants compared to non-stressed plants (Figure 2A,B). On the other hand, chloroplast number per cell in the tissue sections was only slightly increased in the salt-stressed seedlings (Figure 2F). 

### Chloroplast ultrastructure

**Figure 3:**
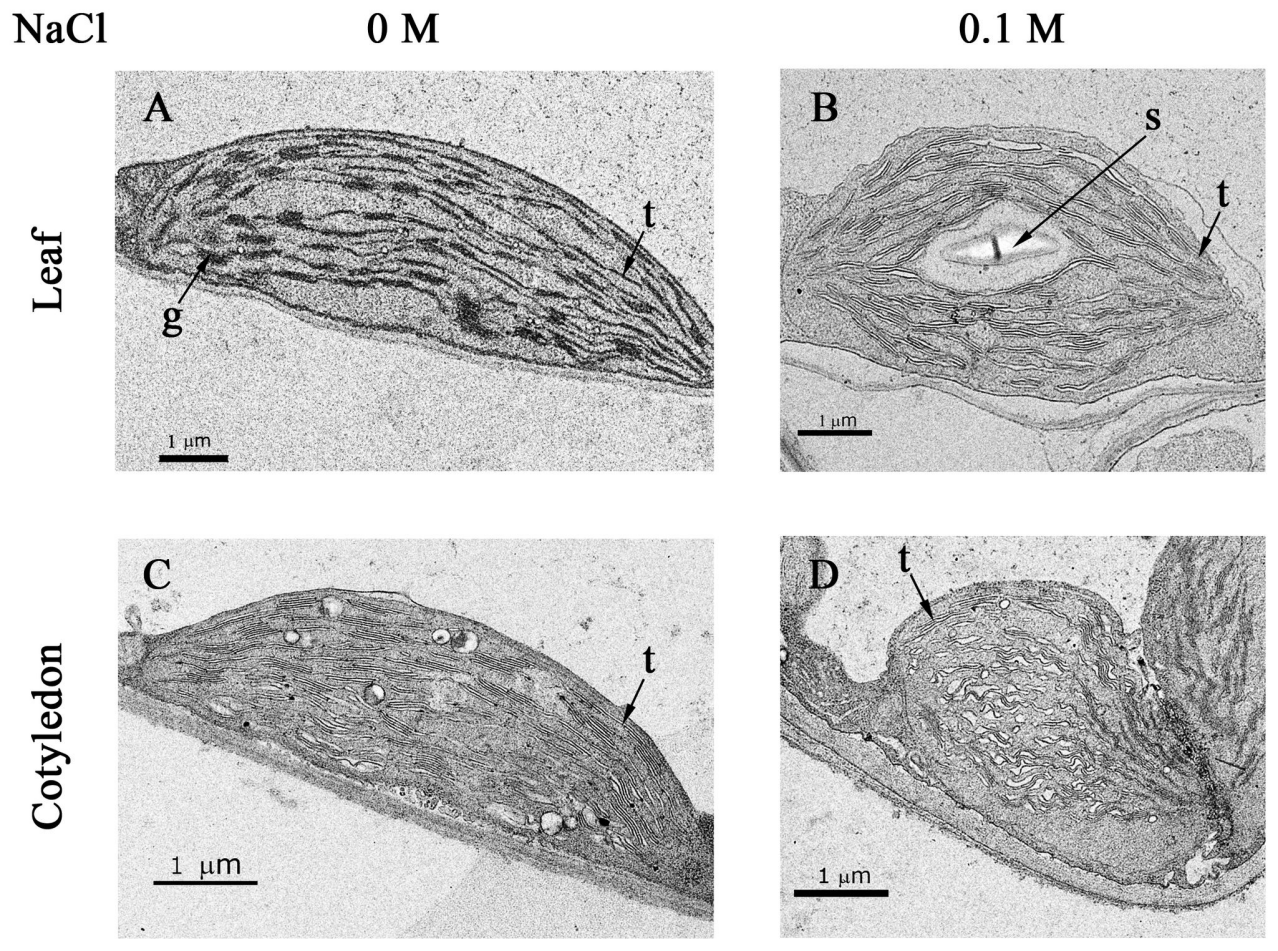
Electron micrographs of chloroplasts from 1^st^ leaves and cotyledons of control and salt-grown seedlings. Biological samples were as described in Figure 2. Ultrathin sections were prepared, stained with uranyl acetate and lead citrate, and examined by transmission electron microscopy. Shown are representative photographs for chloroplasts from (A) 1^st^ leaf of control plant; (B) 1^st^ leaf of salt-grown plant; (C) cotyledon of control plant; (D) cotyledon of salt-grown plant. g, grana; t, thylakoid; s, starch grain.

Comparison of chloroplast ultrastructure using electron microscopy showed that the chloroplasts in leaves of salt-treated seedlings were much less developed than those in control-grown seedlings (Figure 3). Chloroplasts in the leaves of salt-grown plants were larger in size, but their grana contained a markedly reduced number of thylakoid stacks (Figure 3B). In addition, starch grains were observed in the chloroplasts of the salt-grown plant leaves (Figure 3B). Cotyledon chloroplasts are known to have a less developed granum system than leaf chloroplasts (Figure 3C, [16]). Cotyledon chloroplasts of salt-germinated plants were swollen and their inner organization showed further deterioration relative to controls (Figure 3D). 

**Figure 4:**
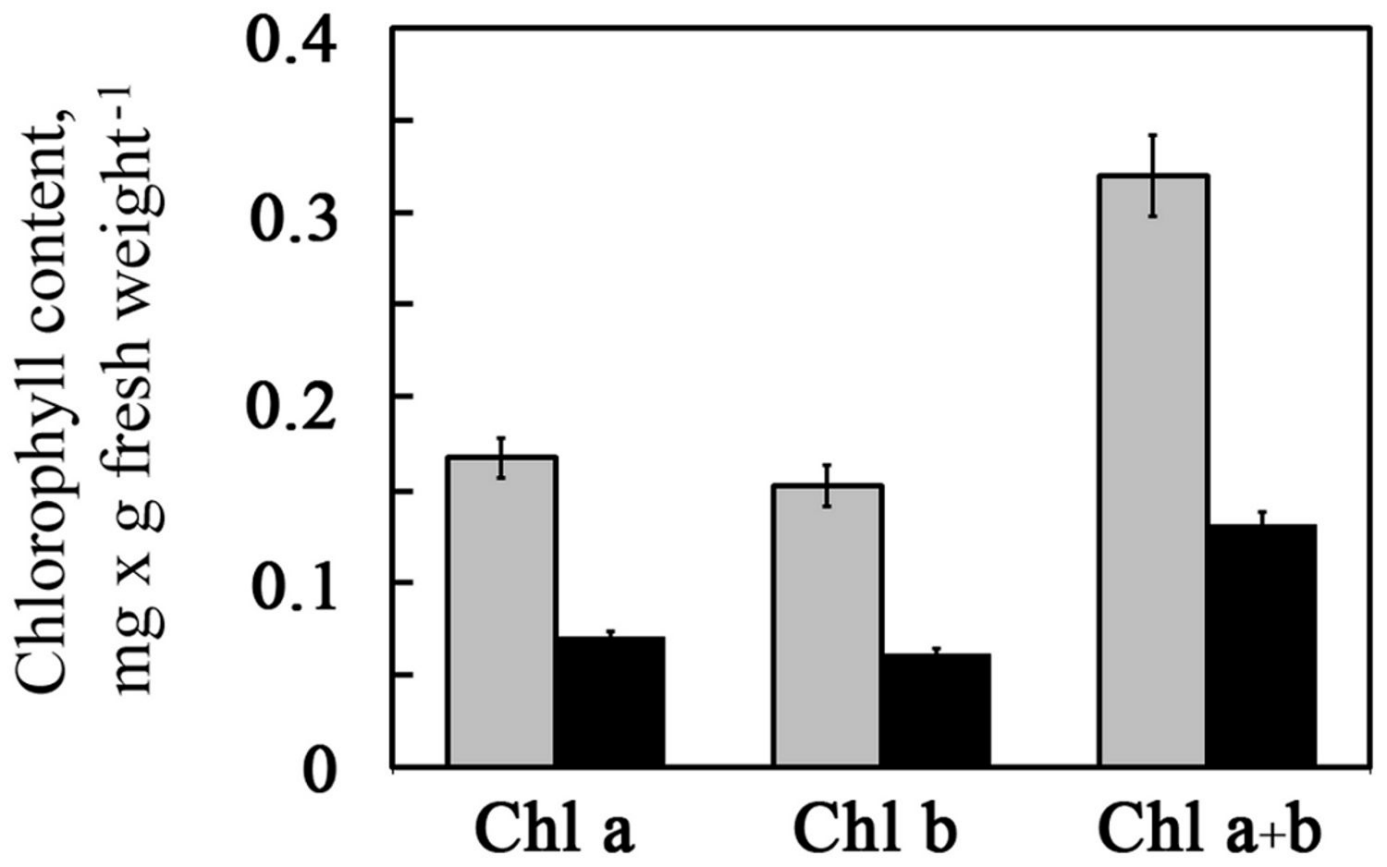
Chlorophyll content of control and salt-grown *Arabidopsis* seedlings. Shoots of 2-week-old *Arabidopsis* seedlings germinated and grown in agar-based medium in the absence (gray bars) or presence (black bars) of 0.1 M NaCl were harvested. Chlorophyll content was assayed as described in Materials and Methods. Data shown are average ± SE.

 Since thylakoid membranes harbor chlorophyll, we determined chlorophyll content in seedlings grown under control and saline conditions. Chlorophyll content in salt-grown seedlings was approximately 40% of that in seedlings grown in the absence of NaCl (Figure 4), with a similar degree of reduction for chlorophyll a and b (Figure 4). This was in agreement with the reduction in granum and thylakoid membranes, which harbor the components of the photosynthetic light reactions, including chlorophyll, in salt-grown seedlings (Figure 3B and D). Reduced chlorophyll content has been measured in *Arabidopsis* [23] and mung bean [24] seedlings germinated and grown in the presence of salt. Reduced chlorophyll content has also been found in *Arabidopsis* seedlings exposed to salt stress [25–27].

In our experimental protocol, NaCl was present throughout all stages of seed germination and seedling development. The results may therefore reflect both plastid/chloroplast duplication and chloroplast development. The number of chloroplasts per mesophyll cell seemed to be only slightly affected by NaCl (Figure 2), suggesting that neither proplastid duplication nor transformation of proplastids to chloroplasts is affected. Proplastids are present mainly in meristem tissues [6,8,28,29]. Moreover, the proplastid–chloroplast transition already occurs in the meristem [9]. 

Our results agree with previous studies showing that chloroplast structure is affected by abiotic stresses, for example swollen chloroplasts in NaCl-treated plants [30] and in those under moderate heat stress (*Arabidopsis*) [31]. Chloroplast structure is impaired in plants exposed to chilling injury [32] and osmotic stress [33]. Impaired chloroplast development has also been observed in plants grown in iron-free or zinc-deficient media [14,34], or in the presence of cadmium [35].

### Transcript levels of chloroplastic and nuclear genes

**Figure 5:**
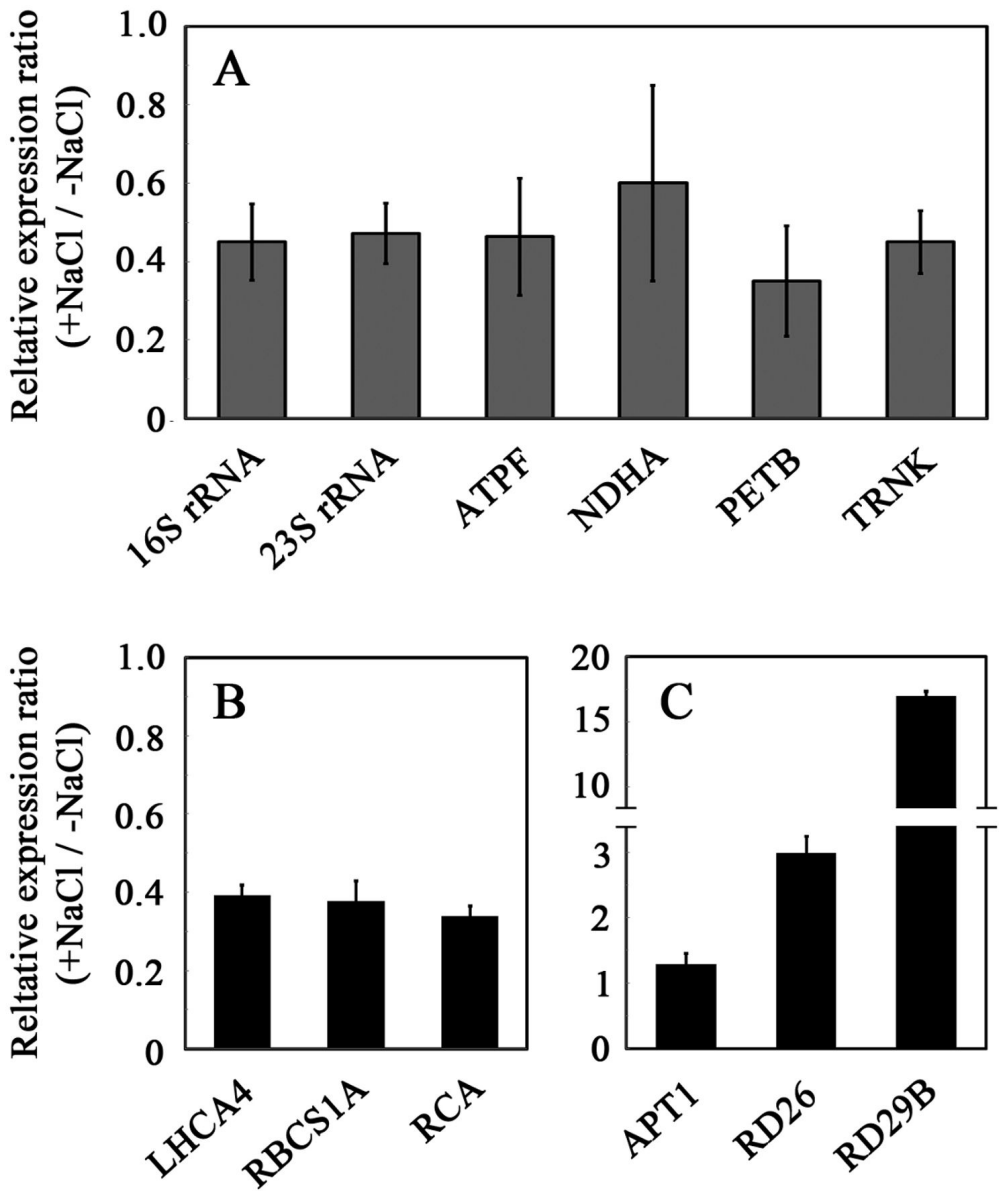
Expression of chloroplast- and nucleus-encoded genes in control and salt-grown seedlings. RNA was prepared from 2-week-old seedlings grown as in Figure 1. cDNA was prepared and the indicated transcript levels were determined by qRT-PCR as described in Materials and Methods using cytosolic 18S rRNA as an internal reference. Steady-state levels of each gene transcript in control grown seedlings were defined as 1. (A) Chloroplast-encoded genes. (B and C) Nucleus-encoded genes. Data shown are average ± SE.

To evaluate the effect of NaCl on transcript levels, we determined the steady-state levels of representative plastid- and nucleus-encoded genes using qRT-PCR. Whereas there is a wealth of studies on the expression of nuclear genes under salt stress, only a handful have studied the effect of salt stress on steady-state transcript levels of plastid-encoded genes. Transcript levels of plastid-encoded genes in salt-stressed seedlings were approximately half of those in non-stressed seedlings (Figure 5A). Both genes involved in plastid translational mechanisms (encoding chloroplast-encoded rRNA and tRNA), and protein-encoding genes were equally affected. As a control, we assayed the steady-state transcript levels of nuclear-encoded genes whose protein products are localized in chloroplasts (Figure 5B). Expression of genes encoding proteins involved in photosynthesis, such as light harvesting (LHCA4) and CO_2_ fixation (RBSC1A and RCA), was also markedly reduced in salt-grown seedlings (Figure 5B). To confirm that the decrease in RNA observed in Figure 5A,B was specific and did not result from global damage to the seedling's transcription ability by the salt treatment, we also assayed nuclear genes whose expression is not altered or induced by exposure to salt. Figure 5C shows that the expression of *APT1* was not affected by salt, whereas transcript levels of *RD26* and *RD29B* encoding salt-modulated transcription factors [36,37] were markedly induced in the salt-grown plants. These results suggest that salt-stress inhibition of photosynthesis-related genes encoded by both plastid and nuclear genomes is specific, and does not seem to result from a global decrease in transcriptional activity in the plant cell. 

### Chloroplast genome copy number

**Figure 6:**
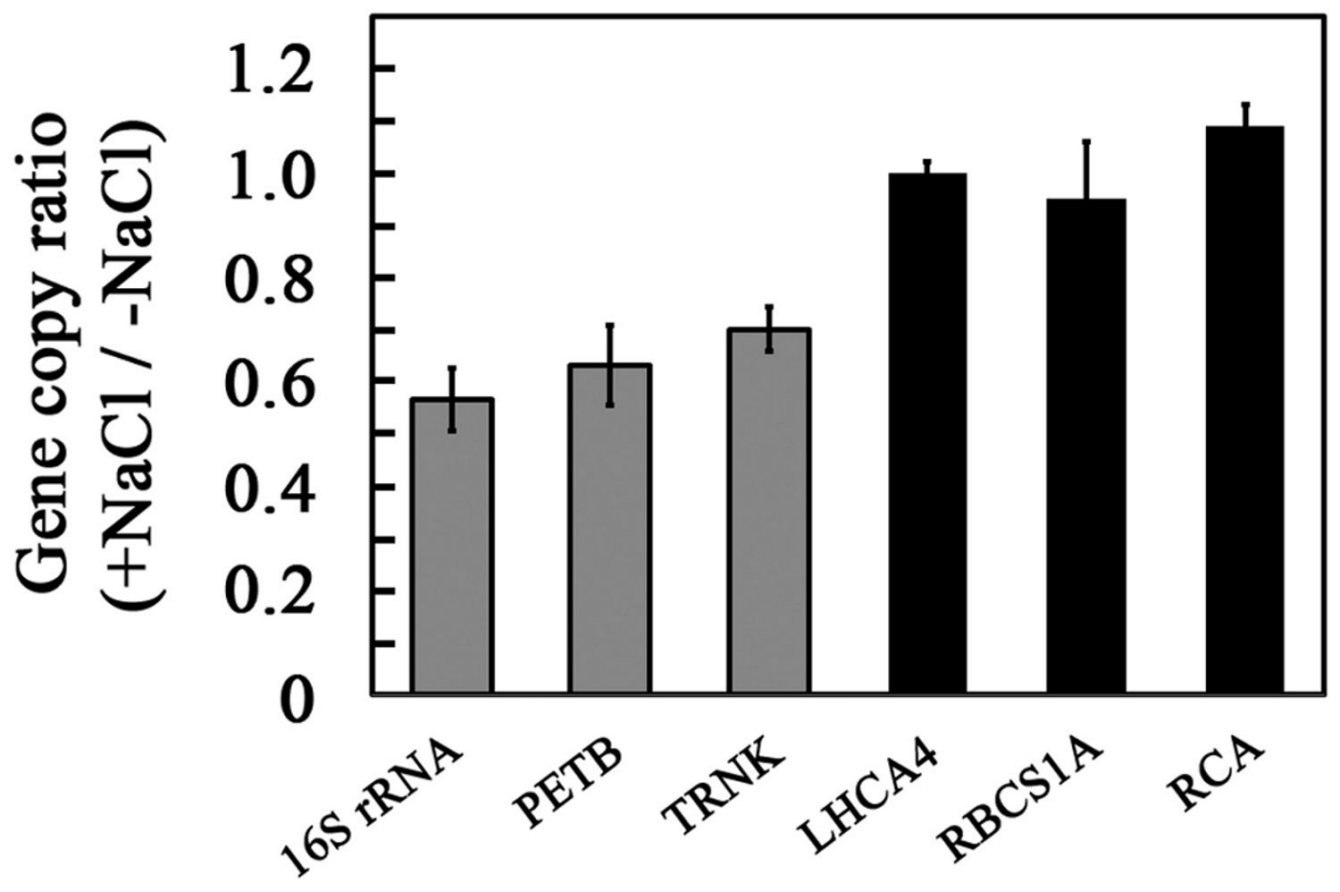
Relative gene copy number in control and salt-grown seedlings. DNA was prepared from 2-week-old seedlings grown as in Figure 1, and used directly for gene-dose analyses by qPCR as described in Materials and Methods. The signal obtained for each gene in control grown seedlings was defined as 1. Gray bars, chloroplast-encoded genes; black bars, nucleus-encoded genes. Data shown are average ± SE.

The observed reduction in steady-state levels of chloroplast-encoded gene transcripts (Figure 5A) might result from a change in the synthesis or degradation of chloroplast RNA, or from a decrease in the copy number of the plastid genome in cells of salt-grown plants. Plastid copy number is known to change in response to developmental changes and environmental signals [14,15]. To determine changes in plastid copy number, we used qPCR (real-time PCR) using DNA templates to compare the template copy ratios between plastid-encoded genes and a single-copy nuclear gene (*WHIRLY 1*). Figure 6 shows that the ratio of chloroplast-to-nuclear genome copy numbers was reduced by about 40% in salt-grown seedlings. As expected, the ratio of nuclear genes encoding chloroplast photosynthetic proteins to the nuclear reference gene was not altered (Figure 6, black bars). Our results suggest that a reduction in genome copy number might be one of the reasons for the decrease in transcript levels of chloroplast-encoded genes. Although chloroplast gene expression is not correlated with genome copy number when comparing different stages of embryogenesis, or cotyledons and leaves of different ages [38,39], these parameters are correlated when looking at leaves of the same age and developmental stage [40]. The molecular mechanism resulting in reduction of chloroplast genome copy number is still to be determined. It was suggested that chloroplast copy number decreases with leaf age due to a lower ratio between the rates replication of plastid chromosomes to chloroplast division [41-44]. On the other hand, the reduction in chloroplast genome copy number in dark grown maize seedlings transferred to light resulted from rapid DNA degradation [45]. 

## Conclusions

We show that *Arabidopsis* seedlings developing under salt-stress conditions are impaired in chloroplast development, and show reduced chloroplast genome copy number, and reduced levels of transcripts of chloroplast-encoded genes and nuclear genes encoding proteins involved in photosynthesis. 

## Supporting Information

File S1 **Supporting tables**.
Table S1. Primers used for qRT-PCR. Table S2. Primers used for qPCR.
(DOC)
