# Supplementary material for: Chloroplasts of Salt-Grown Arabidopsis Seedlings Are Impaired in Structure, Genome Copy Number and Transcript Levels
Source: PLoS One. 2013 Dec 5;8(12):e82548. doi: 10.1371/journal.pone.0082548 (PMC3855474; doi:10.1371/journal.pone.0082548)
Supplement: File S1 — Supporting tables. Table S1. Primers used for qRT-PCR. Table S2. Primers used for qPCR. (DOC) [file pone.0082548.s001.doc]

**Supporting Information**

**Table S1. Primers used for qRT-PCR**

| Primer Sequence | Locus | Gene |
| --- | --- | --- |
|
| AAGCAAGCCTACGCTCTGGA | 18S RRNA (multiple loci) | *18S rRNA* |
| AGGCCAACACAATAGGATCGA |
| AATTCAGAAGAACTGCGTGAAGG | [AtCg01100](http://www.arabidopsis.org/servlets/TairObject?id=1000648029&type=gene) | *ndhA* |
| CCATTCACGCGAAACTTATCC |
| CGTCTTGAGATTCAGGCGATT | AtCg00720 | *petB* |
| AGTAAGGGTAATTCCGCCTAGACA |
| TGCAAGCACGGTTTGGG | AtCg00030 | *trnK* |
| CCGGAACTAGTCGGATGGAGTA |
| GCGCTTGCGTAACGTAGAAAC | AtCg00130 | *atpF* |
| TTTCTCGTTCGATTTCAGAGTATCC |
| GTATCGACCCGTGCAGTGCT | AtCg00920 | *16S rRNA* |
| TTCATTCTTGCGAACGTACTCC | AtCg1210 |
| TGGGCGTTAGAGCATTGAGA | AtCg00950 | *23S rRNA* |
| GGTACACCAGAGGTGCGTCC | AtCg01180 |
| GAACAATGGCTTCCTCTATGCTC | At1g67090 | *RBCS1A* |
| AGCGACCATAGTGGCCTGAG |
| CATGCCTCGGCCTCGAT | At3g47470 | *LHCA4* |
| AACCTACCGGAAGAACCGGT |
| AAACCAACTGGATTATGCACGA | At4g27410 | *RD26* |
| CACAACACCCAATCATCCAACTT |
| GCGCACCAGTGTATGAATCCT | At5g52300 | *RD29B* |
| GCCGGAACATTAGTTTCTCCG |
| AAGGCATATCTGTTGTTGCAGGT | At1g27450 | *APT1* |
| GCACCAATAGCCAACGCAATA |

**Table S2. Primers used for qPCR**

| Primer Sequence | Primer | Gene |
| --- | --- | --- |
|
| AAGCAAGCCTACGCTCTGGA | 18S RRNA (multiple loci) | *18S rRNA* |
| AGGCCAACACAATAGGATCGA |
| GTGAAACGGCATGGATTCG | At1g14410 | *WHIRLY1* |
| TGGCGAGACTTAACCGAGAAGA |
| TGCAAGCACGGTTTGGG | AtCg00030 | *trnK* |
| CCGGAACTAGTCGGATGGAGTA |
| GAGCTTGAGAGTGGAAACGCA | At2g39730 | *RCA (Rubisco activase)* |
| AAGTCAGCTGCCTCACGGTAC |
| GAACAATGGCTTCCTCTATGCTC | At1g67090 | *RBCS1A* |
| AGCGACCATAGTGGCCTGAG |
| CATGCCTCGGCCTCGAT | At3g47470 | *LHCA4* |
| AACCTACCGGAAGAACCGGT |
